# Nanocubosomal based *in situ* gel loaded with natamycin for ocular fungal diseases: development, optimization, *in-vitro*, and *in-vivo* assessment

**DOI:** 10.1080/10717544.2021.1965675

**Published:** 2021-09-13

**Authors:** Khaled M. Hosny, Waleed Y. Rizg, Hala M. Alkhalidi, Walaa A. Abualsunun, Rana B. Bakhaidar, Alshaimaa M. Almehmady, Adel F. Alghaith, Sultan Alshehri, Amani M. El Sisi

**Affiliations:** a Department of Pharmaceutics, Faculty of Pharmacy, King Abdulaziz University, Jeddah, Saudi Arabia; b Center of Excellence for Drug Research and Pharmaceutical Industries, King Abdulaziz University, Jeddah, Saudi Arabia; c Department of Clinical Pharmacy, Faculty of Pharmacy, King Abdulaziz University, Jeddah, Saudi Arabia; d Department of Pharmaceutics, College of Pharmacy, King Saud University, Riyadh, Saudi Arabia; e Department of Pharmaceutics and Industrial Pharmacy, Faculty of Pharmacy, Beni-Suef University, Beni -Suef, Egypt

**Keywords:** Cubosomes, cornea, *in situ* gel, keratitis, natamycin, permeation, phytantriol

## Abstract

Natamycin (NT) is a synthetic broad-spectrum antifungal used in eye drops. However, it has low solubility and high molecular weight, limiting its permeation, and generally causes eye discomfort or irritation when administered. Therefore, the present study aimed to develop an ophthalmic *in situ* gel formulation with NT-loaded cubosomes to enhance ocular permeation, improve antifungal activity, and prolong the retention time within the eye. The NT-loaded cubosome (NT-Cub) formula was first optimized using an I-optimal design utilizing phytantriol, PolyMulse, and NT as the independent formulation factors and particle size, entrapment efficiency %, and inhibition zone as responses. Phytantriol was found to increase particle size and entrapment efficiency %. Higher levels of PolyMulse slightly increased the inhibition zone whereas a decrease in particle size and EE% was observed. Increasing the NT level initially increased the entrapment efficiency % and inhibition zone. The optimized NT-Cub formulation was converted into an *in situ* gel system using 1.5% Carbopol 934. The optimum formula showed a pH-sensitive increase in viscosity, favoring prolonged retention in the eye. The *in vitro* release of NT was found to be 71 ± 4% in simulated tear fluid. The optimum formulation enhanced the *ex vivo* permeation of NT by 3.3 times compared to a commercial formulation and 5.2 times compared to the NT suspension. The *in vivo* ocular irritation test proved that the optimum formulation is less irritating than a commercial formulation of NT. This further implies that the developed formulation produces less ocular irritation and can reduce the required frequency of administration.

## 1. Introduction

Fungal infections, or ophthalmic mycoses, can cause vision loss and even morbidity and present a serious concern worldwide (Rammohan et al., 2020). While eyes have an inherent mechanism to fight against unwarranted organisms and irritants, they are prone to fungal, bacterial, and viral infections due to their characteristic structure and direct contact with the environment (Klotz et al., 2000). Eye infections caused by fungi are less common than those caused by bacteria and viruses and can be, unfortunately, very serious in nature. The pathogenesis of ocular fungal infections is invariably related to epidemiology (Klotz et al., 2000). Particularly, *Candida*, *Aspergillus*, and *Fusarium* are the species responsible for most fungal infections that can occur in many parts of the eye (Thomas, 2003). For instance, keratitis is caused by the infection of the cornea, and endophthalmitis is the infection of the vitreous or aqueous humor (Rammohan et al., 2020). Meanwhile, endophthalmitis can be either exogenous, where the infection is caused by an external source, or endogenous, where the infection spreads from blood (Sheu, 2017).

Polyenes and azoles are the most common types of antifungal agents used in ocular fungal infections. Amphotericin B and natamycin (NT) are widely employed polyenes in eye infections. While amphotericin B is the choice for systemic therapy, it has poor corneal penetration and can even cause corneal discoloration (O'Day et al., 1986; Ansari et al., 2013). Comparatively, NT is less toxic, has better corneal penetration, and, thus, is the first-line drug in fungal keratitis and is considered the best antifungal agent against *Fusarium* and *Aspergillus* (Ansari et al., 2013). Importantly, the US Food & Drug Administration (FDA) approved NT as the first antifungal agent for therapeutic use against fungal keratitis.

As a synthetic broad-spectrum drug against fungal infections, NT is used topically in the form of a 5% suspension dosage form for eye infections. Usually, one drop of this 5% suspension formulation is administered every 1–2 h initially for the first 3–4 days after which the administration frequency can be reduced to 6–8 times a day. However, it has low water solubility (4100 mg/L at 21 °C) and high molecular weight (665.733 g/mol), which limits its corneal permeation, and has been reported to cause eye discomfort or irritation when administered as an eye suspension. Moreover, NT is not suitable for deep infections, and its long-term use is required for successful therapeutic outcomes (Müller et al., 2013). A low NT retention at the surface of the eye and poor bioavailability are serious issues with suspension formulation (Thakkar et al., 2019). Since the drug rapidly drains from the eye, the current commercially available ocular suspension must be administered hourly or every 2 h during the first 3–4 days of treatment. NT can also cause some adverse effects, such as eye irritation, discomfort, or allergies, that are not significant either in systemic or ocular levels (Qiu et al., 2015). In addition to these pharmacological and therapeutic disadvantages, NT also poses pharmaceutical hindrances in product development, including limited aqueous solubility, agglomeration of the suspended powder, and poor chemical stability. Furthermore, its suspension dosage form is vulnerable to bacterial growth and contamination (Patil et al., 2017).

Topical administration is a convenient route for fungal keratitis, while advanced drug delivery approaches can achieve greater corneal permeation and ocular residence (Khames et al., 2019). Various novel therapeutic strategies and approaches for delivering such desired targets have been reported with the progress of nanomedicine and nanotechnology (Xu et al., 2013). Specifically, nanocarriers with a specific surface charge offer several advantages over conventional suspension formulations in ocular therapy (Gorantla et al., 2020). Among the nanocarriers, nanoparticles and liposomes can enhance residence time in the cornea and the bioavailability of NT, while supporting the sustained release of NT, thereby reducing dosing frequency (Patil et al., 2017). Also, nanocarriers decrease toxicity and significantly enhance drug permeation to deeper ocular tissues (Zhou et al., 2013). Several modifications of nanoparticles and liposomes have been performed for various ocular drug delivery applications, such as nanostructured lipid carriers, polymeric nanoparticles, dendrimers, polymeric micelles, solid lipid nanoparticles, cubosomes, etc. Among these, cubosomes have become one of the most sought-after nanostructures for ocular drug delivery due to their exceptional colloidal stability, simple bulk production, and very high internal surface area (Hartnett et al., 2015).

Cubosomes are derived from the lipid cubic phase and become stable in the presence of a polymer-based outer corona. They have a high membrane surface area and can provide better drug loading than liposomes (Barriga et al., 2019). Meanwhile, monoolein and phytantriol are the most common lipids used to prepare cubosomes, whereby phytantriol offers the appropriate safety, biocompatibility, imparting structural stability, and sustained drug release for the use of cubosomes in ocular drug delivery applications (Bessone et al., 2021).

Ocular *in situ* gels are prepared using stimuli-sensitive polymers, which provide a sol-to-gel transformation of the liquid-state dosage form when it is administered to the eye. Polyacrylic acid, poloxamers, sodium alginate, etc. are some of the commonly used stimuli-sensitive polymers that can be used with or without a viscosity modifier, such as hydroxypropyl methylcellulose (Makwana et al., 2016). Increasing the ocular residence is a major advantage of ocular *in situ* gels in addition to providing a sustained drug release. The conversion of cubosomes into an *in situ* gel can combine the advantages of cubosomes and an *in situ* gel; therefore, this combination approach is expected to enhance the ocular permeation and residence time of a drug (Wu et al., 2019).

In the case of NT, some lipid-based nanocarriers have been reported. Niosomes, bilosomes, solid lipid nanoparticles, nanostructured lipid carriers, and transfersomes are prominent among such reported ocular delivery systems for NT (Patil et al., 2018; Janga et al., 2019; Khames et al., 2019). However, the characteristic bicontinuous water channels, separated by the lipid bilayer, in cubosomes facilitate enhanced ocular retention of NT. The cubosomes have the peculiar advantage of the ability to fuse with the cell membrane of the corneal epithelium and acting as an NT reservoir. In addition, cubosomes have high corneal penetration power and stability (Navarro-Partida et al., 2021). Also, the robust internal structure of cubosomes facilitates the accommodation of the drug molecules without significantly increasing the particle size (Chang et al., 2021). All these advantages of cubosome were considered for its selection as the delivery system for NT in the present study. Furthermore, cubosome formulations have been proved beneficial for other drugs too for ocular delivery (Nasr et al., 2020; Elfaky et al., 2021).

Recently, NT-loaded cubosome (using glyceryl monooleate/Span 80) dispersion in poloxamer has been reported for ocular delivery of NT (Kazi et al., 2020). However, phytantriol offers considerably higher structural stability than glyceryl monooleate in the formation of cubosomes (Rizwan et al., 2011). Furthermore, the application of Carbopol ocular drug delivery systems increases the mucoadhesion significantly compared to poloxamer, and that too without affecting the rheological characteristics (Qi et al., 2007). PolyMulse (Acrylates/C10-30 Alkyl Acrylate Crosspolymer) is an emulsifier suitable for the formulation of cubosomes but has not been studied yet. Meanwhile, the addition of hydroxypropyl cellulose (HPC) to carbopol enhances its mucoadhesion and favors drug retention and sustained release (Muthumanikandar et al., 2011). Nevertheless, to date, no studies have been reported to evaluate the use of phytantriol/PolyMulse/Carbopol/HPC cubosome *in situ* gel for ocular delivery of NT. Thus, the present work was aimed to develop an ocular *in situ* gel with optimized cubosomes to increase ocular permeation, improve antifungal action, and prolong the residence time of NT within the eye. This approach was expected to overcome the irritation effect associated with commercially available ocular suspensions and reduce the dosing frequency.

## 2. Materials and methods

### 2.1. Materials

Natamycin was received as a generous gift from the Saudi Arabian Japanese Pharmaceutical Company Limited (SAJA, Jeddah, Saudi Arabia). Phytantriol was purchased from Avanti Polar Lipids (Alabaster, AL, USA). PolyMulse (Acrylates/C10-30 Alkyl Acrylate Crosspolymer) was obtained from Lotioncrafter (Eastsound, Washington, USA). Carbopol 934 and hydroxypropyl cellulose (HPC) were received from Acros Organics (Morris Plains, NJ, USA). Methanol was purchased from Sigma–Aldrich. High-Performance Liquid Chromatography (HPLC) grade solvents were procured from Fisher Scientific (Leicestershire, UK).

### 2.2. Experimental design

The present research followed an I-optimal design using phytantriol (A), PolyMulse (B), and NT (C) as the independent factors. Meanwhile, particle size, entrapment efficiency percentage (EE%), and inhibition zone (a measure of antifungal activity) were the responses. All the selected levels of the independent factors were based on the outcome of the pre-optimization studies. Phytantriol levels were tried based on the quantities mentioned in some reported studies (Lai et al., 2020; Bessone et al., 2021). PolyMulse levels were chosen after the selection of appropriate levels required for the formulation of cubosomes. In the case of NT, the dose of the drug is fixed for the formulations (Thakkar et al., 2019). Nevertheless, it is imperative to study the influence of the NT content on particle size, EE%, and inhibition zone. In the present study, NT quantities 250, 407, 475, 565, 570, 592, 740, or 750 mg were used as specified by the design. However, these quantities had no relation to the dose of NT. The weight of the formulation corresponding to the actual dose of NT can be determined from the drug content. In the case of responses, minimum inhibitory concentration (MIC) can be a better parameter for the assessment of antifungal activity than the zone of inhibition. However, the zone of inhibition can have different values even when the MIC values are the same, as demonstrated in a reported study (Khames et al., 2019). Unfortunately, responses that remain the same and do not change with independent factors are not suitable for inclusion in a design model. So, to have a valid design model, the zone of inhibition is far better than MIC. Furthermore, a change in response is needed for the comparative evaluation of cubosome formulations and optimization. Hence, a zone of inhibition was used in the present study instead of MIC. The details of the design layout (Design-Expert^®^ Version 12 software) are provided in Table 1.

**Table 1.** The independent factors for the design in terms of actual values.

Run	Factor-A	Factor-B	Factor-C
	Phytantriol (mg)	PolyMulse (mg)	Natamycin (mg)
1	1200	200	565
2	1200	600	750
3	1020	592	475
4	1068	330	407
5	1020	592	475
6	992	379	475
7	992	379	475
8	800	200	750
9	948	200	250
10	800	600	750
11	1200	450	250
12	980	481	250
13	1200	440	570
14	800	600	250
15	1200	200	250
16	1020	380	740
17	978	200	592
18	1020	380	740

#### 2.2.1. Natamycin-loaded cubosome (NT-Cub) preparation

Based on the proposed design, various NT-Cub formulations were prepared using 800–1200 mg phytantriol, 200–600 mg PolyMulse, and 250–750 mg NT. During the cubosome preparation, phytantriol in specified quantities was heated in a glass to 50 °C to yield a free-flowing powder. Then, specified quantities of NT and PolyMulse, dissolved in 10 mL pH 5.5 buffer, were added to cubosome glass. Homogenization of this mixture was carried out at 10,000 rpm and 50 °C for 5 min to obtain a milky dispersion. For the preparation of the pH-sensitive *in situ* gel, Carbopol 934 (150 mg) was added to the NT-Cub dispersion (10 mL) with continuous stirring to get a milky dispersion.

#### 2.2.2. Particle size of NT-Cub

The particle size was determined in triplicate (Microtrac^®^ zetatrack particle size analyzer) after diluting the NT-Cub dispersion 10 times with the pH 5.5 buffer.

#### 2.2.3. NT entrapment efficiency percentage

To estimate the entrapment efficiency (EE%) of NT, the prepared cubosome dispersions were centrifuged at 15,000 rpm and 4 °C for 20 min. The supernatant was removed to separate the cubosome pellet, which was then subjected to sonication with methanol (15 min). Finally, the NT content was estimated at 305 nm using a UV–Vis spectrophotometer. The EE% was then determined using Equation (1) (Zhang et al., 2020).

(1) $$\mathrm{EE}\%=\frac{N_{E}}{N_{O}}\times 100$$

where *N_E_* and *N_O_* represent quantities of NT entrapped and originally added, respectively. The value of *N_E_* was determined from the difference between *N_O_* and free NT.

#### 2.2.4. Antifungal activity of NT-Cub

To study the antifungal activity of the NT-loaded cubosomes and optimized NT-Cub, a *Candida albicans* ATCC 76615 strain was tested using the agar well diffusion technique. Briefly, 50 mL Müller–Hinton agar with 1 mL fungal culture, comprising 1 × 10^6^ CFU/mL, into Petri dishes of 150-mm diameter. The fungus was subsequently inoculated, then 200 µL of the prepared NT-Cub formulations were added into holes (12 mm diameter) made in the agar. These were incubated for 4 h at 37 °C. The inhibition zone, areas absent of any fungal growth, was measured by a caliper.

#### 2.2.5. Optimization of NT-Cub formulation

The optimization of the NT-Cub formulation was performed according to the constraints and goals presented in Table 2. The results of the formulation trials were subjected to statistical analysis and numerical optimization of the NT-Cub formulation to achieve the specified goals with respect to the responses. The optimum formula was expected to provide a minimum value for particle size and maximum values for EE% and inhibition zone (Khames et al., 2019).

**Table 2.** Constraints and goals selected to optimize the NT-Cub formulation.

Factor	Name	Goal	Lower limit	Upper limit
Independent factors	Phytantriol	Is in range	800 mg	1200 mg
	PolyMulse	Is in range	200 mg	600 mg
	Natamycin	Is in range	250 mg	750 mg
Responses	Particle size (nm)	Minimize	46 nm	222 nm
	EE (%)	Maximize	51%	91%
	Inhibition zone (mm)	Maximize	9 mm	24.5 mm

### 2.3. Characterization of optimized NT-Cub formulation

#### 2.3.1. Minimum inhibitory concentration (MIC)

The MIC value was determined following a reported and established well diffusion method (Magaldi et al., 2004). The good diffusion studies were carried out using the same method outlined under evaluation of the antifungal activity of NT-Cub in section 2.2.4.

#### 2.3.2. Polydispersity index and zeta potential

The polydispersity index and zeta potential were determined in triplicate (Microtrac^®^ zetatrack particle size analyzer) after diluting the optimized NT-Cub dispersion 10 times with the pH 5.5 buffer.

#### 2.3.3. Drug content

The drug content of NT-Cub was determined following a reported method with slight modification (Patil et al., 2018). Briefly, the NT was extracted from NT-Cub using methanol and the NT content was determined by HPLC at 303 nm using a C18 column and mobile phase prepared with 4 mg/mL ammonium acetate solution: acetonitrile: tetrahydrofuran in a ratio of 76:24:5.

### 2.4. Preparation of NT-loaded cubosomal in situ gel using optimized NT-Cub

The optimized NT-Cub formulation, containing 800 mg phytantriol, 510 mg PolyMulse, and 460 mg NT, was dispersed in Carbopol 934 dispersion (1.5%) for the preparation of the cubosomal *in situ* gel formulation coded as F1. For comparative testing, the optimized NT-Cub formulation was dispersed in phosphate buffer of pH 5.5 without Carbopol 934 and was coded as F2. Also, an NT powder in Carbopol 934 dispersion was prepared and coded as F3.

### 2.5. Characterization of in situ gel (F1) formulation

#### 2.5.1. Rheological properties

The F1 formulation was studied in triplicate using a cone plate rheometer (Brookfield DV III ultra V6.0 RV, Middleboro, MA, USA), and spindle #CPE40 at 25 ± 3 °C. Viscosities of the F1 formulation (0.5 g) before and after gelation were determined. Gelation was achieved by raising the pH to 7.4 by the addition of aqueous sodium hydroxide solution (0.5 M) to the optimized NT-Cub dispersion containing 1.5% Carbopol 934 (Gupta et al., 2010).

#### 2.5.2. *In vitro* release

The NT release from the prepared formulations (F1, F2, and F3) and the aqueous suspension of NT was evaluated in triplicate using the dialysis bag method. A known quantity of each sample (corresponding to 50 mg drug) was kept inside the dialysis bag (molecular weight cutoff 14 k Da, Sigma–Aldrich Inc.). Simulated tear fluid (STF) containing 0.67%w/v sodium chloride, 0.2%w/v sodium bicarbonate, and 0.008%w/v calcium chloride in deionized water and hydrochloric acid (for adjusting pH to 7.4) was prepared. A definite volume of STF was added in a ratio of 25:7 (formulation: STF) to mimic the condition in the human eye. The sample-containing dialysis bags were immersed in glass bottles filled with 250 mL phosphate buffer at pH 7.4 (as the medium), which were kept in a shaking water bath at 34 ± 0.5 °C and 100 rpm (Terreni et al., 2020). Aliquots of 3 mL were taken at time intervals with medium (fresh STF) replacement to maintain sink condition. The concentration of NT in these withdrawn samples was determined at 305 nm by UV–Vis spectrophotometry.

#### 2.5.3. *Ex-vivo* permeation studies

The studies were conducted in triplicate with F1 (optimized NT-Cub dispersed in Carbopol 934 *in situ* gel base), F2 (optimized NT-Cub dispersed in phosphate buffer pH 5.5), NT suspension, and commercial NT (2%) suspension samples. This study aimed to assess the influence of cubosomes in the release behavior and corneal permeation parameters of NT from these samples. The study complied with the ethical principles of the Egyptian Research Institute of Ophthalmology for animal use in experiments. The corneal dissection of albino rabbits was carried out following the reported procedure (Alharbi & Hosny, 2020). The study samples (corresponding to 50 mg drug) were placed in the donor compartment of the diffusion cell, containing phosphate buffer pH 7.4 (7 mL) in the receptor chamber, fitted with the excised rabbit cornea as the barrier membrane. The *ex vivo* permeation samples were withdrawn, then the permeated NT content was determined by HPLC at 303 nm using a C18 column and mobile phase prepared with 4 mg/mL ammonium acetate solution: acetonitrile: tetrahydrofuran in a ratio of 76:24:5. The cumulative amount permeated per unit area was estimated from the withdrawn samples. The diffusion parameters, such as diffusion coefficient (*D*), permeability coefficient (Pc), and steady-state flux (*J*_ss_), were also determined (Alhakamy & Hosny, 2019). The relative permeation rate (RPR) and enhancement factor (EF) were calculated using Equations (2) and (3), respectively:

(2) $$\text{Relative permeation rate }(\mathrm{RPR})=\frac{{\text{Cumulative amount permeated}}_{\left(\mathrm{Test}\right)}}{{\text{Cumulative amount permeated}}_{\left(\text{Commercial product}\right)}}$$

(3) $$\text{Enhancement factor }(\mathrm{EF})\;\frac{{\text{Cumulative amount permeated}}_{\left(\mathrm{Test}\right)}}{{\text{Cumulative amount permeated}}_{\left(\text{Drug suspension}\right)}}$$

#### 2.5.4. *In vivo* ocular irritation test

To examine the eye irritation upon application of the studied formulation, the rabbit eye irritation test was in accordance with a reported procedure (Elfaky et al., 2021). Sixteen healthy New Zealand white rabbits (1.5–2.5 kg) were used. The rabbits were procured from the animal house of Beni-Suef clinical laboratory center, Beni-Suef, Egypt (Approval date: May 2020, Reference No. 35-05-20). The work was conducted in adherence with the Declaration of Helsinki, the International Guiding Principle in Care and Use of Animals (DHEW production NIH 80-23), and the Standards of Laboratory Animal Care (NIH distribution #85-23, reconsidered in 1985). Before starting the experiment, rabbits were adapted for at least two weeks in naturally controlled enclosures at 20 ± 1 °C with a 12/12 h dark/light cycle. Rabbits were randomly classified into five groups, each consisting of three rabbits. Group I received an ocular treatment of 25 µL non-medicated Carbopol 934*in situ* gel formulation (control group). Group II was administered the same volume of F1 formulation (optimized NT-Cub dispersed in Carbopol 934 *in situ* gel base), and Group III was given the same volume of F2 (aqueous dispersion of NT-Cub). Meanwhile, Group IV was given the same volume of F3 (NT powder in Carbopol 934 dispersion), and Group V was administered a commercially available NT product (Pimafucin). The studied formulations were instilled, using a micropipette, into the lower conjunctival sac. The eyes of the treated animals were observed for 8 h. The degree of eye irritation was recorded following the classical Draize test, which was previously described and mentioned by Luechtefeld et al. (2016). The criteria for accepting the ocular irritation test were as follows in Table 3.

**Table 3.** Description of Draize scoring rules.

Endpoint	Description	Range
Cornea	Degree of opacity and ulcerations	0–4
Iris	Swelling, hyperemia	0–2
Conjunctivae	Redness, vessel discernibility	0–3
Chemosis	Swelling, lids closed/open	0–4

## 3. Results and discussion

### 3.1. Experimental design

The results of the responses, including particle size, EE%, and inhibition zone, for each experimental run are provided in Table 4.

**Table 4.** The independent factors and responses for the design of experiments.

Run	Factor-A	Factor-B	Factor-C	Response 1		Response 2		Response 3	
	Phytantriol (mg)	PolyMulse (mg)	Natamycin (mg)	Particle size (nm)		EE (%)		Inhibition Zone (mm)	
				Observed	Predicted	Observed	Predicted	Observed	Predicted
1	1200	200	565	222	222.46	91	91.28	11.5	11.49
2	1200	600	750	173	173.99	59	59.88	13	13.26
3	1020	592	475	153	152.81	84	85.18	18	17.93
4	1068	330	407	167	168.02	87	85.68	16	16.04
5	1020	592	475	154	152.81	85	85.18	18	17.93
6	992	379	475	74	75.61	80	81.76	24.5	24.64
7	992	379	475	76	75.61	81	81.76	24.5	24.64
8	800	200	750	143	142.79	53	52.44	13	12.87
9	948	200	250	174	172.96	70	68.86	11.5	11.16
10	800	600	750	64	65.54	50	49.59	21.5	21.44
11	1200	450	250	160	159.91	67	67.67	14	14.06
12	980	481	250	115	116.87	65	64.08	16	16.17
13	1200	440	570	169	167.95	89	85.80	18	17.55
14	800	600	250	45	45.45	62	62.84	22	21.94
15	1200	200	250	212	211.65	72	73.38	9	9.14
16	1020	380	740	147	147.75	55	56.89	13.5	13.72
17	978	200	592	200	201.07	83	82.83	11.5	11.80
18	1020	380	740	150	147.75	57	56.89	14	13.72

#### 3.1.1. Effect on particle size

A quadratic response surface model was suggested for particle size with a *p*-value of <.0001 and a lack of fit *p*-value of 0.4412. Thus, for the particle size response, the quadratic response surface model was acceptable with a non-significant lack of fit *p*-value. Further, the predicted (0.9966) and adjusted R-squared (0.9991) values were close. Also, the fitted particle size was in good agreement with the observed particle size (Table 3).

The analysis of variance (ANOVA) data for the particle size of NT-Cub formulations is provided in Table 5. From the results, it can be seen that A, B, C, AB, AC, BC, A^2^, B^2^, and C^2^ had significant influences on the particle size of NT-Cub.

**Table 5.** ANOVA data for particle size of NT-Cub formulations of experimental runs.

Source	Sum of squares	Degrees of freedom	Mean square	*F*-value	*p*-Value	–
Model	43,271.08	9	4807.90	2207.74	<.0001	Significant
A-Phytantriol	22,999.97	1	22,999.97	10,561.36	<.0001	–
B-PolyMulse	6882.07	1	6882.07	3160.18	<.0001	–
C-Natamycin	299.04	1	299.04	137.32	<.0001	–
AB	433.46	1	433.46	199.04	<.0001	–
AC	194.06	1	194.06	89.11	<.0001	–
BC	17.97	1	17.97	8.25	.0208	–
A²	2082.34	1	2082.34	956.19	<.0001	–
B²	3814.68	1	3814.68	1751.66	<.0001	–
C²	320.36	1	320.36	147.11	<.0001	–
Residual	17.42	8	2.18	–	–	–
Lack of fit	11.92	5	2.38	1.30	.4412	Not significant
Pure error	5.50	3	1.83	–	–	–
Cor total	43288.50	17	–	–	–	–

**Figure 1.**
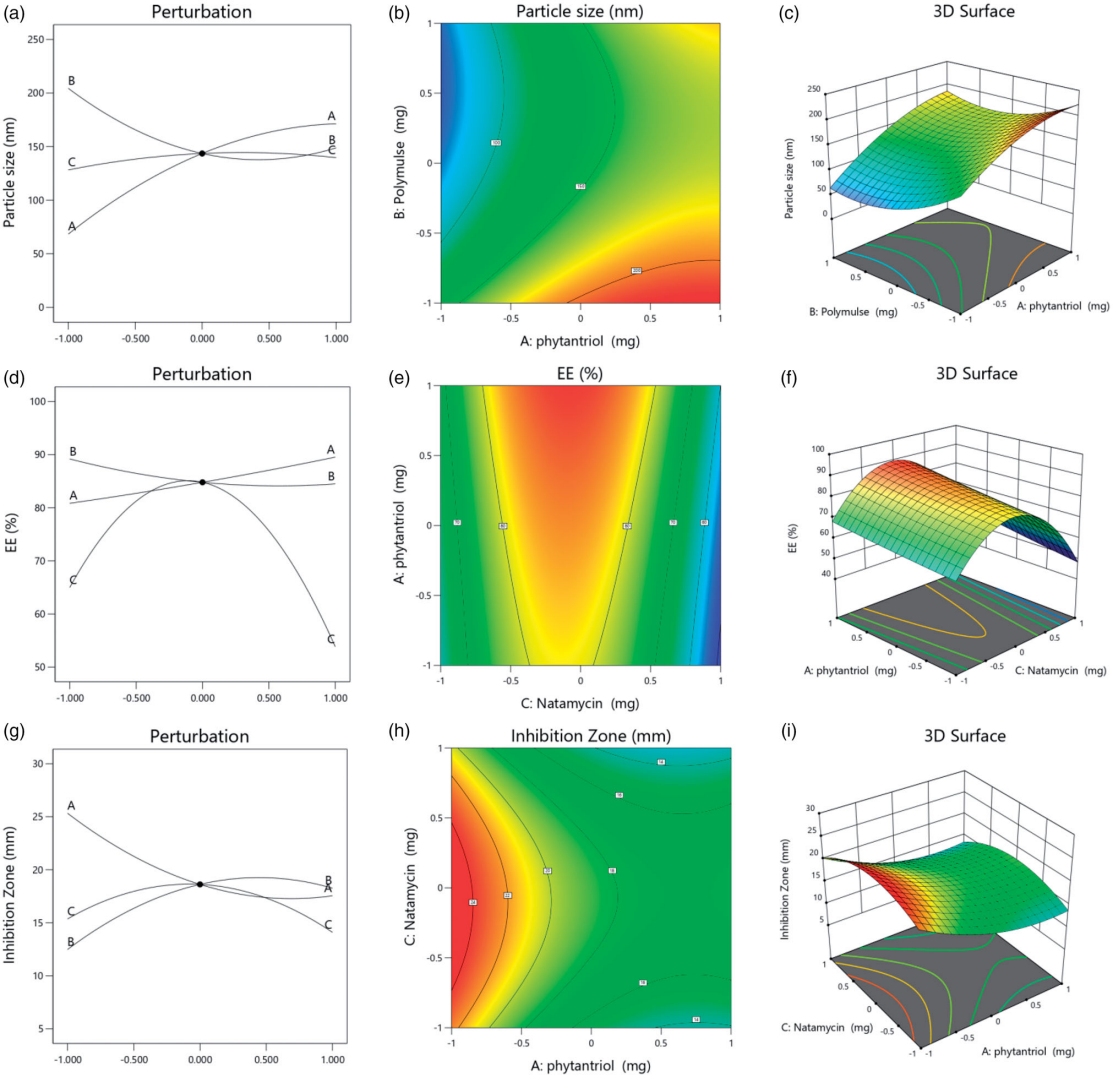
Design plots of the various responses of NT-Cub formulations: (a) perturbation plot for particle size, (b) contour plot for particle size, (c) response surface plot for particle size, (d) perturbation plot for EE, (e) contour plot for EE, (f) response surface plot for EE, (g) perturbation plot for inhibition zone, (h) contour plot for inhibition zone, and (i) response surface plot for inhibition zone.

The equation suggested (in terms of coded factors) by the Design-Expert^®^ Version 12 software for particle size of NT-Cub formulations is provided in Equation (4). From the equation and the ANOVA data, it can be observed that all independent factors (terms A, B, and C) had a significant influence on the particle size of the NT-Cub formulations. Among these factors, phytantriol (term A) had the highest influence on particle size, as indicated by the highest coefficient value, while natamycin quantity had the least influence. The polynomial equation and the perturbation plot (Figure 1(a)) indicate that the particle size increased as the amount of phytantriol in the formulation increased. This can be attributed to the reduced shearing effects and a greater possibility of cubosome aggregation at higher phytantriol concentrations (Alharbi & Hosny, 2020). On the contrary, the equation and perturbation plot (Figure 1(a)) suggest that higher quantities of PolyMulse in the formulation reduced the particle size. PolyMulse is a polymeric emulsifier that acts as a stabilizer in the formation of cubosomes, thereby an increase in the polymeric stabilizer imparts higher interfacial stability and reduces the aggregation of cubosomes. Such an increase in particle size of cubosomes by the poloxamer polymer as a stabilizer has also been reported (Luechtefeld et al., 2016). Nevertheless, excessive concentration of a stabilizer can have no or adverse effect on particle size by excessively interfering with the structure of the phytantriol cubosome membrane. This might be the reason for the slight increase in particle size of the cubosomes noted at higher values of PolyMulse in the respective perturbation plot (Figure 1(a)). Another interesting observation is that NT quantity showed a much smaller effect on cubosome particle size, where only a very slight increase in particle size occurred with the increasing NT level. While higher drug quantities typically result in larger nanoparticles and liposomes, cubosomes have a robust internal structure to accommodate the drug molecules without significantly increasing the particle size. This specific advantage of cubosomes has been established with curcumin (Chang et al., 2021). The particle size was also found to be dependent on other terms, namely AB, AC, BC, A^2^, B^2^, and C^2^. Therefore, the net influence on the particle size will be different from the individual effects because of these interaction effects. The contour and response surface plots of the net effect on cubosome particle size are shown in Figures 1(b,c), respectively. These plots reveal increased particle size at higher levels of phytantriol and decreased particle size at higher levels of PolyMulse, yet neither phytantriol nor PolyMulse was found to influence each other in terms of particle size.

(4) $$\text{Particle}\;\text{size}\;=\;+143.63\;+51.17A-27.64B+5.75C+9.17\text{AB}-6.11\;\text{AC}-1.81\text{BC}-23.{51A}^{2}+32.{97\;B}^{2}-9.{62C}^{2}$$

#### 3.1.2. Effect on entrapment efficiency percentage (EE%)

A quadratic response surface model, with a *p*-value of <.0001, was suggested for the EE% by the software. Further, the lack of fit was not significant with a *p*-value >.05. Thus, the quadratic response surface model was acceptable for the EE% response, and the predicted and adjusted R-squared values of 0.9171 and 0.9805, respectively, were comparable. Moreover, the fitted values for EE% were found to have an adequate correlation with the observed values (Table 3).

The ANOVA data for the EE of NT-Cub formulations in Table 6 show that A, B, C, and C^2^ are significant model terms with a significant influence on EE%.

**Table 6.** ANOVA data for EE% of NT-Cub formulations of experimental runs.

Source	Sum of squares	Degrees of freedom	Mean square	*F*-value	*p*-Value	–
Model	3068.65	9	340.96	95.85	<.0001	Significant
A-Phytantriol	153.32	1	153.32	43.10	.0002	–
B-PolyMulse	43.12	1	43.12	12.12	.0083	–
C-Natamycin	276.87	1	276.87	77.83	<.0001	–
AB	1.00	1	1.00	0.2825	.6095	–
AC	10.38	1	10.38	2.92	.1261	–
BC	0.5529	1	0.5529	0.1554	.7037	–
A²	1.01	1	1.01	0.2840	.6086	–
B²	16.37	1	16.37	4.60	.0642	–
C²	2195.55	1	2195.55	617.20	<.0001	–
Residual	28.46	8	3.56	–	–	–
Lack of fit	25.46	5	5.09	5.09	.1054	Not significant
Pure error	3.00	3	1.0000	–	–	
Cor total	3097.11	17	–	–	–	–

The polynomial equation (coded factors) suggested by the software for EE% of NT-Cub formulations is provided in Equation (5). The effect of individual factors is shown in the perturbation plot obtained for EE% (Figure 1(d)). The results indicate that higher levels of phytantriol favor a slight increase in EE%, which was also observed in phytantriol cubosomes with ciprofloxacin (Alharbi & Hosny, 2020). This result can be attributed to the enhanced rigidity of the lipid bilayer due to higher phytantriol, possible formation of multilayered membranes in vesicles, and high affinity of lipophilic drugs toward phytantriol (Perugini & Pavanetto, 1998; El-Nabarawi et al., 2013). Specifically, EE% slightly decreased with increasing levels of PolyMulse, which may be due to the presence of polymeric stabilizers that cause shifting of the phase boundaries or changes in the phase behavior of the lipids (Barriga et al., 2019). Thus, changes produced by the inclusion of PolyMulse may result in the drug-holding capacity of the lipid membrane. The perturbation plot shows that EE% first increased then later decreased at higher polymer levels. This effect is justified by the significant effects of the terms C and C^2^, as suggested by the ANOVA data. It may be assumed that at lower levels, the phytantriol content of cubosomes was sufficient to accommodate the increasing amounts of NT. However, higher NT levels reduced the possibility of accommodation in lipid layers and further decreased EE%. A previous study on colchicine-loaded cubosomes demonstrated that greater drug concentrations increased EE% at higher lipid concentrations and decreased EE% at lower lipid concentrations (Nasr et al., 2020). Thus, the argument that higher drug concentrations reduce the effective concentration of lipids available for drug entrapment is justified. The contour (Figure 1(e)) and response surface (Figure 1(f)) plots for EE% show that NT has the highest influence on EE% and phytantriol has the least effect. Also, these plots show that EE% first increased then later decreased as NT levels increased.

(5) $$\text{EE}\;=\;+84.65\;+4.18A-2.19B-5.53C-0.4414\text{AB}\;+1.41\;\text{AC}\;+0.3184\;\text{BC}+0.5178A^{2}+2.16B^{2}-25.19C^{2}$$

#### 3.1.3. Effect on inhibition zone

The suggested model (quadratic) for the inhibition zone had a *p*-value <.0001 and a lack of fit *p*-value of 0.2067 and, thus, was acceptable for the evaluation and optimization of the NT-Cub formulation. The predicted and adjusted R-squared values were 0.9772 and 0.9958, respectively, which are close (Table 4). The ANOVA data for the inhibition zone is shown in Table 7, where A, B, C, AB, AC, BC, A^2^, B^2^, and C^2^ are significant model terms influencing the inhibition zone.

**Table 7.** ANOVA data for inhibition zone of NT-Cub formulations of experimental runs.

Source	Sum of squares	Degrees of freedom	Mean square	*F*-value	*p*-Value	–
Model	363.90	9	40.43	446.48	<.0001	Significant
A-phytantriol	133.07	1	133.07	1469.42	<.0001	–
B-PolyMulse	75.30	1	75.30	831.45	<.0001	–
C-Natamycin	3.61	1	3.61	39.89	.0002	–
AB	1.88	1	1.88	20.80	.0018	–
AC	0.8607	1	0.8607	9.50	.0150	–
BC	3.38	1	3.38	37.37	.0003	–
A²	29.96	1	29.96	330.81	<.0001	–
B²	37.02	1	37.02	408.82	<.0001	–
C²	52.28	1	52.28	577.34	<.0001	–
Residual	0.7245	8	0.0906	–	–	–
Lack of fit	0.5995	5	0.1199	2.88	.2067	Not significant
Pure error	0.1250	3	0.0417	–	–	–
Cor total	364.63	17	–	–	–	–

Equation (6) represents the polynomial equation suggested by the software for the inhibition zone in terms of coded factors. The perturbation plot in Figure 1(g) shows that the inhibition zone has a significant influence on all independent factors (A, B, and C). Initially, the inhibition zone drastically decreased as the level of phytantriol increased then plateaued with greater phytantriol levels. The high affinity of lipophilic drugs toward phytantriol and enhanced rigidity of the lipid bilayer due to higher phytantriol content might have contributed to less NT release from the cubosomes, finally resulting in a decrease in the inhibition zone (Perugini & Pavanetto, 1998; El-Nabarawi et al., 2013; Luechtefeld et al., 2016). Nevertheless, some studies have reported enhanced antimicrobial activity of drugs in the cubosome formulation against *Pseudomonas* (Lai et al., 2020). Meanwhile, the perturbation plot shows that higher PolyMulse levels slightly increased the inhibition zone, which might be due to the increased drug release by the polymer that altered the phase behavior of lipids and reduced the drug-holding capacity (Barriga et al., 2019). The effect of NT levels on the inhibition zone is similar to that on EE% but to a lesser extent. Initially, increasing the NT level slightly increased the inhibition zone then later decreased it, possibly due to the influence of NT on EE%. Specifically, as EE% increased, more NT was loaded into the NT-Cub and became available for release, thus increasing the inhibition zone. In other words, at higher initial EE% values, the inhibition zone increased then later decreased as EE% was reduced. The contour (Figure 1(h)) and response surface (Figure 1(i)) plot also confirm these results of the inhibition zone.

(6) $$\begin{array}{l} \text{Inhibition}\;\text{zone}=+18.62\;-3.89\text{ A }\\ +2.89\text{ B }-0.6321\text{ C }-0.6044\;\text{AB}\;+0.4069\text{AC}+0.7876\text{BC}\;+2.{82\;A}^{2}-\;3.{25B}^{2}-3.{89C}^{2} \end{array}$$

#### 3.1.4. Optimization of cubosome formulation

The optimum formula and the responses, both actual and predicted, are provided in Table 8. The optimized formula was found to be comprised of 800 mg phytantriol, 510 mg PolyMulse, and 460 mg NT.

**Table 8.** Optimum formula, the predicted responses, and the observed responses for the NT-Cub formulation.

Independent factors			
Factor	Name	Level	Actual value
A	Phytantriol	−1.0000	800 mg
B	PolyMulse	0.5604	510 mg
C	Natamycin	−0.1861	460 mg

Responses		
	Predicted value	Observed value
Particle size (nm)	55.5352	57 nm
EE (%)	80.6291	82%
Inhibition zone (mm)	26.2479	25.5 mm

**Table 9.** *Ex vivo* permeation data of natamycin (NT) from F1 (optimized NT-Cub dispersed in Carbopol 934 *in situ* gel base), F2 (optimized NT-Cub dispersed in phosphate buffer pH 5.5), NT suspension, and commercial formulation (Pimafucin) samples.

Parameters of permeation	F1	F2	NT suspension	Commercial formulation
Cumulative amount permeated (μg/cm^2^)	1613 ± 210^†,#,$^	1101 ± 166^@,#,$^	310 ± 91^@,†,$^	488 ± 68^@,†,#^
Steady-state flux, *J*_ss_ (μg/cm^2^·min)	11.221	8.227	2.833	3.644
Permeability coefficient, Pc (cm/min)	6.4 × 10^−4^	4.8 × 10^−4^	1.9 × 10^−4^	2.5 × 10^−4^
Diffusion coefficient, *D* (cm^2^/min)	20.2 × 10^−4^	15.6 × 10^−4^	5.6 × 10^−4^	7.1 × 10^−4^
Relative permeation rate (RPR)	3.305	2.256	0.635	–
Enhancement factor (EF)	5.203	3.551	–	1.574

Statistical inferences: ^@^*p* < .05, compared with F1; ^†^*p* < .05, compared with F2; ^#^*p* < .05, compared with NT suspension; ^$^*p* < .05, compared with commercial formulation.

### 3.2. Characterization of optimized NT-Cub formulation

The MIC value for the optimized NT-Cub formulation was found to be 0.15 mcg/ml Meanwhile, the polydispersity index and zeta potential were found to be 0.3 and 22 mV, respectively. The drug content was found to be 0.93%.

### 3.3. Preparation and characterization of NT-loaded cubosomal in situ gel using optimized NT-Cub

Formulations F1 (optimized NT-Cub dispersed in Carbopol 934 *in situ* gel base), F2 (optimized NT-Cub dispersed in phosphate buffer pH 5.5), and F3 (NT powder dispersed in Carbopol 934 *in situ* gel base) were prepared and evaluated.

#### 3.3.1. Rheological properties

To confirm the *in situ* gelling behavior of the F1 formulation prepared with buffer at pH 5.5, the viscosities were determined before and after raising the pH to 7.4. The viscosity of the F1 formulation increased from 875 ± 34 to 3213 ± 434 cp as the pH increased from 5.5 to 7.4, respectively. Previous studies have well-described the rheological properties of Carbopol 934 dispersions and a concentration-dependent enhancement in gel viscosity in the case of ocular *in situ* gels with Carbopol 934 (Barry & Meyer, 1979; Abou El Ela & Khatib, 2014). It can be assumed that the enhancement in viscosity of the F1 formulation at pH 7.4 would help to increase the retention time of the NT drug in the eye. Such increase in retention time in ocular delivery by viscosity enhances has been clearly identified in previous reports (Dubashynskaya et al., 2019).

#### 3.3.2. *In vitro* release

**Figure 2.**
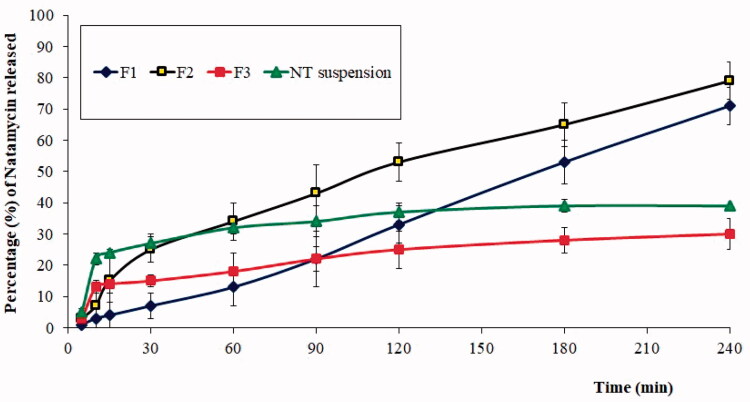
*In vitro* drug release profiles of natamycin (NT) from F1 (optimized NT-Cub dispersed in Carbopol 934 *in situ* gel base), F2 (optimized NT-Cub dispersed in phosphate buffer pH 5.5), F3 (NT powder dispersed in Carbopol 934 *in situ* gel base), and NT suspension samples.

The *in vitro* NT release from the *in situ* gelling F1 formulations in comparison to F2, F3, and NT suspension samples is shown in Figure 2. The F1 formulation released 71 ± 4% of the drug after 4 h compared to 79 ± 8% from F2, and without any statistically significant difference (*p* < .05). Meanwhile, the F3 formulation and NT suspension showed lower NT releases of 30 ± 3 and 39 ± 1%, respectively, after 4 h. It can be seen that F1 and F2 provide a sustained release of NT, which agrees with a previous study that reported sustained drug release by cubosomes (El-Enin & AL-Shanbari, 2018). Interestingly, there was no burst release from the F1 and F2 formulations, also agreeing with reported studies that show drug release without a burst effect from cubosomes (Luechtefeld et al., 2016). The observed results further confirm that the NT-Cub formulation can significantly increase drug release compared to the pure drug. The ability of cubosomes to enhance the dissolution of poorly water-soluble drugs has resulted in such an observation (Mei et al., 2017). At the same time, a smaller NT release from F1 compared to F2 at initial sampling points can be attributed to the higher viscosity of the F1 formulation due to the pH 7.4 STF used in the *in vitro* release study. This can be further justified by the observed NT release profiles for F3 and NT suspension, where the only difference was the presence of Carbopol 934 *in situ* gel base in F3. Moreover, the release of NT from F3 was lower than that from the NT suspension. However, the *in vitro* of NT observed in the present study was higher than that from the previously reported NT-loaded glyceryl monooleate/Span 80 cubosome dispersion (Kazi et al., 2020). The reported study observed around 85% release only after 8 h, but with an initial burst release. The reported study was carried out in phosphate buffer pH 7.4 and poloxamer was used as the gelling agent. These might have produced the difference observed for *in vitro* release. Interestingly, NT-loaded solid lipid nanoparticles also had a biphasic release pattern with an initial burst (Khames et al., 2019). Thus, avoidance of burst release and a steady sustained release profile can be highlighted as a characteristic advantage of the F1 formulation. Meanwhile, the *in vitro* NT release from reported niosomes also was devoid of burst phase and thus was comparable to F1 formulation (El-Nabarawi et al., 2019).

#### 3.3.3. *Ex vivo* permeation studies

The *ex vivo* permeation study results showed a significant difference (*p* > .05) between the percent permeated from F1 compared to all other tested samples (Table 9). The flux of corneal permeation of NT followed the order: F1 > F2 > NT suspension > commercial NT (2%) suspension. The F1 formulation enhanced the permeation of NT 3.3 times compared to the commercially available product and 5.2 times compared to the NT suspension. This observation is supported by reports that demonstrated enhanced corneal permeation and bioavailability of cubosomes (Han et al., 2010; ElMeshad & Mohsen, 2016). Another work proposed that the absorption and/or surface lipid exchange between cubosomes and cells in the epithelium of the cornea are responsible for the enhanced corneal permeation by cubosomes (Han et al., 2010). In particular, the *in situ* gelling behavior of Carbopol 934 enhances the adhesion and intimate contact between the drug and corneal membrane (Khare et al., 2014). Thus, the F1 formulation prepared by incorporation of NT-Cub within *in situ* gel base containing Carbopol 934 enhanced the *ex vivo* permeation of NT compared to the NT-Cub aqueous dispersion. Nevertheless, the enhancement of NT permeation was less than that reported from transfersome-loaded gellan *in situ* gel wherein around 6- to 9-folds enhancement was noted (Janga et al., 2019). Similarly, the reported NT-loaded glyceryl monooleate/Span 80 cubosome dispersion also showed enhanced permeation than from a suspension formulation (Kazi et al., 2020). Meanwhile, the permeability coefficient and steady-state flux were found to be higher from NT-loaded solid lipid nanoparticles compared to pure NT (Khames et al., 2019). Thus, the F1 formulation was comparable to these systems in terms of corneal permeation enhancement of NT. Meanwhile, the permeability of NT from nanostructured lipid carriers was only around 4-folds compared to a commercial suspension formulation (Patil et al., 2018). Thus, it can be seen that the F1 formulation is better in the enhancement of corneal permeation than the nanostructured lipid carrier.

#### 3.3.4. *In vivo* ocular irritation test

Table 10 presents the results of eye irritation tests conducted on New Zealand white rabbits with the prepared NT-Cub formula. Conjunctival edema (chemosis), redness in the conjunctiva, secretion, corneal opacity, and iris involvement was taken as the evaluation parameters. Scores for each parameter and total scores obtained for the formulations were considered in the study.

**Table 10.** Scores obtained from eye irritation assessment of different formulations tested on New Zealand white rabbits.

Lesion	Score for each lesion	Control group (non-medicated Carbopol 934 *in situ* gel formulation)	F1 (optimized NT-Cub dispersed in Carbopol 934 *in situ* gel base)	F2 (aqueous dispersion of NT-Cub)	F3 (NT powder in Carbopol 934 dispersion)	Commercially available NT aqueous suspension
(A) Conjunctival edema (chemosis)						
No swelling	0	0	–	–	–	–
Any swelling	1	–	1	1	1	1
Prominent swelling along with partial lid eversion	2	–	–	–	–	–
Swelling with half-closed lids	3	–	–	–	–	–
Swelling with totally closed lids	4	–	–	–	–	–
(B) Redness in conjunctiva						
Absent	0	0	0	–	–	–
Abnormal conjunctival injections	1	–	–	1	–	1
More diffuse and deeper hyperemia, separate vessels cannot be seen easily	2	–	–	–	2	–
Diffuse and dense hyperemia	3	–	–	–	–	–
(C) Secretion						
Absent	0	–	–	0	–	–
Any abnormal secretion	1	1	1		1	1
Secretion leading to wet eye lashes closer to lids	2	–	–	–	–	–
Secretion leading to wet lids and whole periorbital area	3	–	–	–	–	–
(D) Corneal opacity						
Absent	0	0	0	0	–	–
Scattered or diffuse areas, detail of the iris discernible	1	–	–	–	1	1
Easy discernible, transparent areas, detail of the iris slightly darkened	2	–	–	–	–	–
Opalescent areas, no details of the iris discernible, size of the pupil barely discernible	3	–	–	–	–	–
Opaque cornea, iris not discernible	4	–	–	–	–	–
(E) Iris involvement						
Absent	0	0	0	0		
Pronounced deep folds, congestion, deep swelling circumcorneal injection, the iris still reacts to light	1	–	–	–	1	1
No response, hemorrhage, marked destruction	2	–	–	–	–	–
	Total score	1	2	2	6	5

In the case of conjunctival edema (chemosis), all samples showed a score of 1 except the control group, which showed no swelling and had a score of zero. This confirms that Carbopol 934 dispersion at a concentration of 1.5% produced no conjunctival edema. No redness in conjunctiva was caused by the control and F1 formulation groups, which both scored zero for this parameter. Meanwhile, animals administered with the F2 formulation and the commercially available NT aqueous suspension had a score of 1. The redness in the conjunctiva was highest for the F3 formulation group with a score of 2, which might be due to the presence of larger NT drug particles. Comparatively, the commercially available NT aqueous suspension may contain sufficiently smaller NT drug particles to avoid such irritation.

All formulations containing Carbopol 934 (including the control) and the commercially available NT aqueous suspension produced some abnormal secretion and had a score of 1. This indicated that the presence of Carbopol 934 polymer causes mild abnormal secretion. The commercially available NT aqueous suspension might also contain Carbopol or a similar thickening agent or mucoadhesive polymer to cause slight irritation. Meanwhile, the F2 formulation with NT-Cub alone did not show any secretion, indicating the absence of any such irritation potency of NT-Cub. Cubosomes are generally non-irritant agents for ophthalmic drug delivery, and this might be the reason for this observation (Han et al., 2010). Corneal opacity was absent in the control, F1, and F2 groups, while F3 and the commercially available NT aqueous suspension had scores of 1 for corneal opacity. The presence of NT in the particulate form might explain the increase in the corneal opacity of these samples. The results of iris involvement were the same as that observed for corneal opacity, where F3 and the commercially available NT aqueous suspension had scores of 1. The total score showed that the NT-Cub formulations (F1 and F2) caused significantly less ocular irritation than F3 and the commercially available NT aqueous suspension. Such lower ocular irritancy in comparison with commercial products has been reported with solid lipid nanoparticles as well (Li et al., 2011). Moreover, there was no change in ocular irritation when NT-Cub was converted into the Carbopol 934 *in situ* gel (F1). Overall, the non-irritancy of ocular formulations containing Carbopol polymer is well-established (Gilhotra et al., 2011). Similar results were observed with the previously reported NT-loaded glyceryl monooleate/Span 80 cubosome dispersion and NT-loaded niosome hydrogel (El-Nabarawi et al., 2019; Kazi et al., 2020).

## 4. Conclusions

In summary, an I-optimal design was employed to optimize NT-Cub formulation for *in situ* gel for ophthalmic delivery. The quantities of phytantriol, PolyMulse, and NT were taken as the independent factors, while particle size, EE%, and inhibition zone were evaluated as responses. All the independent factors were found to significantly influence these responses. The optimized NT-Cub formulation was found to contain 800 mg phytantriol, 510 mg PolyMulse, and 460 mg NT. Subsequently, the optimized NT-Cub formulation was converted into an *in situ* gel system using 1.5% Carbopol 934 dispersion in pH 5.5 buffer (F1) then further evaluated. The viscosity of the formulation was significantly increased when pH was increased to 7.4. The *in vitro* NT release from the F1 and F2 formulations was similar after 4 h and significantly higher than that from F3 and NT suspension. The *ex vivo* permeation studies confirmed higher drug permeation from cubosomes, and the flux of corneal permeation of NT followed the order F1 > F2 > NT suspension > commercial NT (2%) suspension. Further, the *in vivo* ocular irritation test indicates that the cubosome formulations produce less ocular irritation than a commercial formulation. Overall, the findings of the study confirm that the F1 formulation can reduce ocular irritancy, enhance corneal permeation, and prolong the residence time of NT.

## References

[CIT0001] Abou El Ela AESF, Khatib MME. (2014). Formulation and evaluation of new long acting metoprolol tartrate ophthalmic gels. Saudi Pharm J 22:555–63. 25561869 10.1016/j.jsps.2014.03.003 PMC4281596

[CIT0002] Alhakamy NA, Hosny KM. (2019). Nano-vesicular delivery system loaded by bifonazole: preparation, optimization, and assessment of pharmacokinetic and antifungal activity. J Drug Deliv Sci Technol 49:316–22.

[CIT0003] Alharbi WS, Hosny KM. (2020). Development and optimization of ocular *in situ* gels loaded with ciprofloxacin cubic liquid crystalline nanoparticles. J Drug Deliv Sci Technol 57:101710.

[CIT0004] Ansari Z, Miller D, Galor A. (2013). Current thoughts in fungal keratitis: diagnosis and treatment. Curr Fungal Infect Rep 7:209–18. 24040467 10.1007/s12281-013-0150-110.1007/s12281-013-0150-1 PMC3768010

[CIT0005] Barriga HMG, Holme MN, Stevens MM. (2019). Cubosomes: the next generation of smart lipid nanoparticles? Angew Chem Int Ed Engl 58:2958–78. 29926520 10.1002/anie.201804067 PMC6606436

[CIT0006] Barry BW, Meyer MC. (1979). The rheological properties of carbopol gels I. Continuous shear and creep properties of carbopol gels. Int J Pharm 2:1–25. 10.1111/j.2042-7158.1974.tb10164.x 4156725

[CIT0007] Bessone CDV, Akhlaghi SP, Tártara LI, et al. (2021). Latanoprost-loaded phytantriol cubosomes for the treatment of glaucoma. Eur J Pharm Sci 160:105748. 33567324 10.1016/j.ejps.2021.105748

[CIT0008] Chang C, Meikle TG, Drummond CJ, et al. (2021). Comparison of cubosomes and liposomes for the encapsulation and delivery of curcumin. Soft Matter 17:3306–13. 33623948 10.1039/d0sm01655a

[CIT0009] Dubashynskaya N, Poshina D, Raik S, et al. (2019). Polysaccharides in ocular drug delivery. Pharmaceutics 12:22. 10.3390/pharmaceutics12010022 PMC7023054 31878298

[CIT0010] El-Enin HA, AL-Shanbari AH. (2018). Nanostructured liquid crystalline formulation as a remarkable new drug delivery system of anti-epileptic drugs for treating children patients. Saudi Pharm J 26:790–800. 30202219 10.1016/j.jsps.2018.04.004 PMC6128721

[CIT0011] Elfaky MA, Sirwi A, Tolba HH, et al. (2021). Development, optimization, and antifungal assessment of ocular gel loaded with ketoconazole cubic liquid crystalline nanoparticles. J Pharm Sci 110:2210–20. 33621518 10.1016/j.xphs.2021.02.022

[CIT0012] ElMeshad AN, Mohsen AM. (2016). Enhanced corneal permeation and antimycotic activity of itraconazole against *Candida albicans* via a novel nanosystem vesicle. Drug Deliv 23:2115–23. 25080226 10.3109/10717544.2014.942811

[CIT0013] El-Nabarawi MA, Abd El Rehem RT, Teaima M, Abary M, et al. (2019). Natamycin niosomes as a promising ocular nanosized delivery system with ketorolac tromethamine for dual effects for treatment of candida rabbit keratitis; *in vitro*/*in vivo* and histopathological studies. Drug Dev Ind Pharm 45:922–36. 30744431 10.1080/03639045.2019.1579827

[CIT0014] El-Nabarawi MA, Bendas ER, El Rehem RTA, Abary MYS. (2013). Transdermal drug delivery of paroxetine through lipid-vesicular formulation to augment its bioavailability. Int J Pharm 443:307–17. 23337629 10.1016/j.ijpharm.2013.01.016

[CIT0015] Gilhotra RM, Nagpal K, Mishra DN. (2011). Azithromycin novel drug delivery system for ocular application. Int J Pharm Investig 1:22–8. 10.4103/2230-973X.76725 PMC3465116 23071916

[CIT0016] Gorantla S, Rapalli VK, Waghule T, et al. (2020). Nanocarriers for ocular drug delivery: current status and translational opportunity. RSC Adv 10:27835–55. 35516960 10.1039/d0ra04971a PMC9055630

[CIT0017] Gupta S, Samanta MK, Raichur AM. (2010). Dual-drug delivery system based on *in situ* gel-forming nanosuspension of forskolin to enhance antiglaucoma efficacy. AAPS PharmSciTech 11:322–35. 20182824 10.1208/s12249-010-9388-x PMC2850500

[CIT0018] Han S, Shen J, Gan Y, et al. (2010). Novel vehicle based on cubosomes for ophthalmic delivery of flurbiprofen with low irritancy and high bioavailability. Acta Pharmacol Sin 31:990–8. 20686524 10.1038/aps.2010.98 PMC4007820

[CIT0019] Hartnett TE, O'Connor AJ, Ladewig K. (2015). Cubosomes and other potential ocular drug delivery vehicles for macromolecular therapeutics. Expert Opin Drug Deliv 12:1513–26. 25745885 10.1517/17425247.2015.1021680

[CIT0020] Janga KY, Tatke A, Balguri SP, et al. (2018). Ion-sensitive *in situ* hydrogels of natamycin bilosomes for enhanced and prolonged ocular pharmacotherapy: *in vitro* permeability, cytotoxicity and *in vivo* evaluation. Artif Cells Nanomed Biotechnol 46:1039–50. 29475386 10.1080/21691401.2018.1443117 PMC6148389

[CIT0021] Janga KY, Tatke A, Dudhipala N, et al. (2019). Gellan gum based sol-to-gel transforming system of natamycin transfersomes improves topical ocular delivery. J Pharmacol Exp Ther 370:814–22. 30872389 10.1124/jpet.119.256446 PMC6806353

[CIT0022] Kazi M, Dhakne R, Dehghan MH. (2020). Ocular delivery of natamycin based on monoolein/span 80/poloxamer 407 nanocarriers for the effectual treatment of fungal keratitis. JRP 24:251–63.

[CIT0023] Khames A, Khaleel MA, El-Badawy MF, El-Nezhawy AOH. (2019). Natamycin solid lipid nanoparticles – sustained ocular delivery system of higher corneal penetration against deep fungal keratitis: preparation and optimization. Int J Nanomedicine 14:2515–31. 31040672 10.2147/IJN.S190502 PMC6459158

[CIT0024] Khare A, Grover K, Pawar P, Singh I. (2014). Mucoadhesive polymers for enhancing retention in ocular drug delivery: a critical review. Rev Adhes Adhesives 2:467–502.

[CIT0025] Klotz SA, Penn CC, Negvesky GJ, Butrus SI. (2000). Fungal and parasitic infections of the eye. Clin Microbiol Rev 13:662–85. 11023963 10.1128/cmr.13.4.662-685.2000 PMC88956

[CIT0026] Lai X, Ding Y, Wu C-M, et al. (2020). Phytantriol-based cubosome formulation as an antimicrobial against lipopolysaccharide-deficient gram-negative bacteria. ACS Appl Mater Interfaces 12:44485–98. 32942850 10.1021/acsami.0c13309

[CIT0027] Li R, Jiang S, Liu D, et al. (2011). A potential new therapeutic system for glaucoma: solid lipid nanoparticles containing methazolamide. J Microencapsul 28:134–41. 21142697 10.3109/02652048.2010.539304

[CIT0028] Luechtefeld T, Maertens A, Russo DP, et al. (2016). Analysis of Draize eye irritation testing and its prediction by mining publicly available 2008–2014 REACH data. ALTEX 33:123–34. 26863293 10.14573/altex.1510053 PMC5461467

[CIT0029] Magaldi S, Mata-Essayag S, Hartung de Capriles C, et al. (2004). Well diffusion for antifungal susceptibility testing. Int J Infect Dis 8:39–45. 14690779 10.1016/j.ijid.2003.03.002

[CIT0030] Makwana SB, Patel VA, Parmar SJ. (2016). Development and characterization of *in-situ* gel for ophthalmic formulation containing ciprofloxacin hydrochloride. Results Pharma Sci 6:1–6. 26949596 10.1016/j.rinphs.2015.06.001 PMC4760229

[CIT0031] Mei L, Xie Y, Jing H, et al. (2017). A novel design for stable self-assembly cubosome precursor-microparticles enhancing dissolution of insoluble drugs. Drug Dev Ind Pharm 43:1239–43. 28276277 10.1080/03639045.2017.1304958

[CIT0032] Müller G, Kara-José N, Castro R. (2013). Antifungals in eye infections: drugs and routes of administration. Rev Brasoftalmol 72:132–41.

[CIT0033] Muthumanikandar R, Edavalath S, Robert JH, et al. (2011). Development and *in-vitro* evaluation of buccoadhesive tablets of losartan potassium. Int J Drug Deliv 3:465–71.

[CIT0034] Nasr M, Teiama M, Ismail A, et al. (2020). *In vitro* and *in vivo* evaluation of cubosomal nanoparticles as an ocular delivery system for fluconazole in treatment of keratomycosis. Drug Deliv Transl Res 10:1841–52. 32779112 10.1007/s13346-020-00830-4

[CIT0035] Nasr M, Younes H, Abdel-Rashid RS. (2020). Formulation and evaluation of cubosomes containing colchicine for transdermal delivery. Drug Deliv Transl Res 10:1302–13. 32399604 10.1007/s13346-020-00785-6

[CIT0036] Navarro-Partida J, Castro-Castaneda CR, Santa Cruz-Pavlovich FJ, et al. (2021). Lipid-based nanocarriers as topical drug delivery systems for intraocular diseases. Pharmaceutics 13(5):678. 10.3390/pharmaceutics13050678 PMC8151015 34065059

[CIT0037] O'Day DM, Head WS, Robinson RD, Clanton JA. (1986). Corneal penetration of topical amphotericin B and natamycin. Curr Eye Res 5:877–82. 3490954 10.3109/02713688609029240

[CIT0038] Patil A, Lakhani P, Majumdar S. (2017). Current perspectives on natamycin in ocular fungal infections. J Drug Deliv Sci Technol 41:206–12.

[CIT0039] Patil A, Lakhani P, Taskar P, et al. (2018). Formulation development, optimization, and *in vitro*-*in vivo* characterization of natamycin-loaded PEGylated nano-lipid carriers for ocular applications. J Pharm Sci 107:2160–71. 29698725 10.1016/j.xphs.2018.04.014

[CIT0040] Perugini P, Pavanetto F. (1998). Liposomes containing boronophenylalanine for boron neutron capture therapy. J Microencapsul 15:473–83. 9651869 10.3109/02652049809006874

[CIT0041] Qi H, Chen W, Huang C, et al. (2007). Development of a poloxamer analogs/carbopol-based *in situ* gelling and mucoadhesive ophthalmic delivery system for puerarin. Int J Pharm 337:178–87. 17254725 10.1016/j.ijpharm.2006.12.038

[CIT0042] Qiu S, Zhao G-Q, Lin J, et al. (2015). Natamycin in the treatment of fungal keratitis: a systematic review and meta-analysis. Int J Ophthalmol 8:597–602. 26086015 10.3980/j.issn.2222-3959.2015.03.29 PMC4458670

[CIT0043] Rammohan R, Suneetha V, Sen S, et al. (2020). Fungal infections of the eye. Curr Clin Micro Rep 7:39–50.

[CIT0044] Rizwan SB, Assmus D, Boehnke A, et al. (2011). Preparation of phytantriol cubosomes by solvent precursor dilution for the delivery of protein vaccines. Eur J Pharm Biopharm 79:15–22. 21237267 10.1016/j.ejpb.2010.12.034

[CIT0045] Sheu SJ. (2017). Endophthalmitis. Korean J Ophthalmol 31:283–9. 28752698 10.3341/kjo.2017.0036 PMC5540982

[CIT0046] Terreni E, Chetoni P, Tampucci S, et al. (2020). Assembling surfactants-mucoadhesive polymer nanomicelles (ASMP-nano) for ocular delivery of cyclosporine-A. Pharmaceutics 12:253. 10.3390/pharmaceutics12030253 PMC7150936 32168973

[CIT0047] Thakkar R, Patil A, Mehraj T, et al. (2019). Updates in ocular antifungal pharmacotherapy: formulation and clinical perspectives. Curr Fungal Infect Rep 13:45–58.

[CIT0048] Thomas PA. (2003). Fungal infections of the cornea. Eye 17:852–62. 14631389 10.1038/sj.eye.6700557

[CIT0049] Wu Y, Liu Y, Li X, et al. (2019). Research progress of *in-situ* gelling ophthalmic drug delivery system. Asian J Pharm Sci 14:1–15. 32104434 10.1016/j.ajps.2018.04.008 PMC7032175

[CIT0050] Xu Q, Kambhampati SP, Kannan RM. (2013). Nanotechnology approaches for ocular drug delivery. Middle East Afr J Ophthalmol 20:26–37. 23580849 10.4103/0974-9233.106384 PMC3617524

[CIT0051] Zhang L, Li J, Tian D, et al. (2020). Theranostic combinatorial drug-loaded coated cubosomes for enhanced targeting and efficacy against cancer cells. Cell Death Dis 11:1. 31911576 10.1038/s41419-019-2182-0 PMC6946659

[CIT0052] Zhou H-Y, Hao J-L, Wang S, et al. (2013). Nanoparticles in the ocular drug delivery. Int J Ophthalmol 6:390–6. 23826539 10.3980/j.issn.2222-3959.2013.03.25 PMC3693026

